# Role of Sodium-Glucose Co-Transporter 2 Inhibitors in the Regulation of Inflammatory Processes in Animal Models

**DOI:** 10.3390/ijms23105634

**Published:** 2022-05-18

**Authors:** Sandra Feijóo-Bandín, Alana Aragón-Herrera, Manuel Otero-Santiago, Laura Anido-Varela, Sandra Moraña-Fernández, Estefanía Tarazón, Esther Roselló-Lletí, Manuel Portolés, Oreste Gualillo, José Ramón González-Juanatey, Francisca Lago

**Affiliations:** 1 Cellular and Molecular Cardiology Research Unit, Institute of Biomedical Research (IDIS) and Xerencia de Xestión Integrada de Santiago de Compostela (XXIS/SERGAS), 15706 Santiago de Compostela, Spain; alana.aragon.herrera@sergas.es (A.A.-H.); leumanleuman@hotmail.com (M.O.-S.); laura.anido.varela@sergas.es (L.A.-V.); sandra.morana.fernandez@usc.es (S.M.-F.); jose.ramon.gonzalez.juanatey@sergas.es (J.R.G.-J.); francisca.lago.paz@sergas.es (F.L.); 2 Centro de Investigación Biomédica en Red de Enfermedades Cardiovasculares (CIBERCV), Institute of Health Carlos III, 28029 Madrid, Spain; tarazon_est@gva.es (E.T.); esther_rosello_lleti@hotmail.com (E.R.-L.); portoles_man@gva.es (M.P.); 3 Cardiology Group, Center for Research in Molecular Medicine and Chronic Diseases (CIMUS), Universidade de Santiago de Compostela, 15706 Santiago de Compostela, Spain; 4 Cardiocirculatory Unit, Health Research Institute of La Fe University Hospital, 46026 Valencia, Spain; 5 Laboratory of Neuroendocrine Interactions in Rheumatology and Inflammatory Diseases, Institute of Biomedical Research (IDIS) and Xerencia de Xestión Integrada de Santiago de Compostela (XXIS/SERGAS), 15706 Santiago de Compostela, Spain; oreste.gualillo@sergas.es

**Keywords:** SGLT2i, inflammation, M1/M2 macrophages, NLRP3 inflammasome, pyroptosis, metaflammation, cytokines

## Abstract

Sodium-glucose co-transporter 2 inhibitors, also known as gliflozins, were developed as a novel class of anti-diabetic agents that promote glycosuria through the prevention of glucose reabsorption in the proximal tubule by sodium-glucose co-transporter 2. Beyond the regulation of glucose homeostasis, they resulted as being effective in different clinical trials in patients with heart failure, showing a strong cardio-renal protective effect in diabetic, but also in non-diabetic patients, which highlights the possible existence of other mechanisms through which gliflozins could be exerting their action. So far, different gliflozins have been approved for their therapeutic use in T2DM, heart failure, and diabetic kidney disease in different countries, all of them being diseases that have in common a deregulation of the inflammatory process associated with the pathology, which perpetuates and worsens the disease. This inflammatory deregulation has been observed in many other diseases, which led the scientific community to have a growing interest in the understanding of the biological processes that lead to or control inflammation deregulation in order to be able to identify potential therapeutic targets that could revert this situation and contribute to the amelioration of the disease. In this line, recent studies showed that gliflozins also act as an anti-inflammatory drug, and have been proposed as a useful strategy to treat other diseases linked to inflammation in addition to cardio-renal diseases, such as diabetes, obesity, atherosclerosis, or non-alcoholic fatty liver disease. In this work, we will review recent studies regarding the role of the main sodium-glucose co-transporter 2 inhibitors in the control of inflammation.

## 1. Introduction

Since the discovery of phlorizin as a natural glucose-lowering molecule with anti-diabetic properties [1,2], many efforts have been made to develop new anti-diabetic drugs based on its chemical structure while improving its bioavailability, stability, and biological function [3,4]. 

Phlorizin (phloretin-2-’O-β-D-glucopyranoside), also referred to as phloridzin, is an O-glucoside of phloretin, a member of the dihydrochalcone family that is, in turn, a subclass of flavonoids [5]. It was originally isolated from the bark of the apple tree and proposed as a promising drug to treat malaria, fever, and infectious diseases due to its bitter taste, similar to extracts from the cinchona and willow tree, which had known antipyretic properties [6,7]. Many decades later, von Mering described for the first time its glucosuric effect and associated reduction of glycemia [8]. Phlorizin’s main pharmacological action is to block renal and intestinal glucose absorption through the non-selective inhibition of both sodium-glucose co-transporters 1 and 2 (SGLT1/2) to promote glucose excretion, which in turn reduces circulating glucose levels and improves insulin resistance [1]. More recently, other effects of phlorizin have been described using different in vivo and in vitro approaches, including cardioprotection, neuroprotection, the improvement of non-alcoholic fatty liver disease (NAFLD) and hepatic metabolism, body weight reduction, or the regulation of inflammation, fibrosis, and oxidative stress (Table 1).

Based on the structure of phlorizin, other synthetic SGLT2 inhibitors (SGLT2i) have been developed in the last decade as therapeutic agents to treat type-2 diabetes mellitus (T2DM), which have also been proved to have beneficial effects beyond glucose lowering [9]. However, despite most of them having been approved for their use in different countries, the exact mechanism through which they exert their actions besides glycosuria is not fully understood so far. Anyway, it seems clear that the development of SGLT2i has supposed a considerable improvement in the treatment of T2DM, heart failure, and diabetic renal disease [10]. 

Inflammation is a protective response of the immune system to engage extreme alterations in homeostasis due to infection, toxic compounds, damaged cells, or irradiation, in which cells in the innate immune system, adaptive immune system, and inflammatory mediators promote the removal of the harmful stimuli and the initiation of the healing process [11,12]. Although its activation is vital to health, when a homeostatic disturbance persists over time it can lead to non-resolving chronic inflammation which creates a vicious circle that perpetuates and worsens the pathological state, such as, for example, adipose tissue inflammation in obesity, lipotoxicity, or hyperglycemia [11]. The deregulation of the inflammatory response is a well-known factor associated with numerous diseases, among them T2DM, heart failure, or diabetic renal disease, which are the main diseases for which the use of SGLT2i has been approved so far [13]. An understanding of the mechanisms that regulate the deregulation of the inflammatory response in pathological conditions is crucial to be able to elaborate more accurate therapeutic approaches aimed at the modulation of inflammation. Indeed, inflammation has been suggested as a potential therapeutic target of SGLT2i [14]. Thus, in this work, we will make an overview of the main SGLT2i developed and discuss its potential use to treat inflammatory processes according to the main publications on the topic indexed in the databases PubMed, Scopus, and Web of Science.

**Table 1:** Main phlorizin’s effects described in the bibliography beyond glycosuria and the improvement of insulin resistance.

Effect	Tissue/Cell Type	Animal Model
Anti-inflammatory	Bladder	Diabetic Akita mice [15]
	RAW 264.7 macrophages [16]	-
	3T3-L1 cells differentiated into adipocytes [17]	-
	Brain Serum, liver, and cecum Gastrointestinal tract Brain Plasma	Aged mice [18] High-fat diet-fed mice [19] Rats with irritable bowel syndrome [20] Mice with Alzheimer’s disease [21] Ovariectomized rats under inflammation conditions [22]
Body weight reduction	-	Diabetic *db/db* mice [23,24] High-fat diet-fed mice [19,25]
Lipolysis	3T3-L1 cells differentiated into adipocytes [16]	-
Anti-oxidant	Brain and liver	Aged mice [18]
	PC12 cells [18]	-
	Skeletal muscle	Mice with exercise-induced fatigue [26]
	HepG2 cells [27]	-
	Liver Serum, liver, and cecum	Rats with hepatic fibrosis [28] High-fat diet-fed mice [19]
Renoprotective	Kidney	Diabetic *db/db* mice [23]
Hepatoprotective	Liver	Diabetic mice with NAFLD [29]
	Liver	Rats with hepatic fibrosis [28]
	Liver	High-fat diet-fed mice [19]
Regulation of gut microbiota homeostasis	Gut microbiota Gut microbiota	High-fat diet-fed mice [19,25] Diabetic *db/db* mice [24]
Bone homeostasis	MC3T3-E1 cells differentiated into osteoblasts and in bone Bone	SAM mice [30] Ovariectomized rats under inflammation conditions [22]
Improvement of cognitive function	Brain Brain	Mice with Alzheimer’s disease [21] Swiss mice [31]
Anti-cancer	KYSE450 and KYSE30 cell lines [32]	-
Cardioprotective	Heart ACBRI 5118 cells [33] HUVEC cells [34] Heart	Diabetic *db/db* mice [35] - Guinea Pig [36]

RAW 264.7: a macrophage-like, Abelson leukemia virus-transformed cell line derived from BALB/c mice; 3T3-L1: fibroblast cell line isolated from the embryo of a mouse; *db/db*: monogenic, insulin-resistant model of T2DM due to a spontaneous mutation in the leptin receptor; PC12: cell line derived from a transplantable rat pheochromocytoma; HepG2: cell line exhibiting epithelial-like morphology that was isolated from a hepatocellular carcinoma of a 15-year-old, white, male youth with liver cancer; MC3T3-E1: osteoblastic cell line established from a C57BL/6 mouse calvaria; SAM: senescence-accelerated mice; KYSE450 and KYSE30: human esophageal cancer cells; ACBRI 5118: primary human cardiac fibroblast cells.

## 2. SGLT1/2 Biology 

The human solute carrier family 5 (SLC5) is made up of 12 members, which mainly transport small molecules such as carbohydrates, vitamins, amino acids, and organic ions such as choline or short-chain fatty acids across cell membranes [37]. Among them, SGLT1 and SGLT2, encoded by the genes SLC5A1 and SLC5A2, are the most studied due to their known association with congenital glucose–galactose malabsorption and familial renal glycosuria, respectively, when they are defective [38,39], and to their recent role as therapeutic targets for treating T2DM.

SGLT1/2 are transmembrane proteins that bind sodium at the extracellular surface. This opens a gate to trap outside sugar and transport both molecules into the cell by flipping the co-transport, and then flipping back to the original conformation to start over a cycle that occurs ∼1000 times/s at a physiological temperature [40]. SGLT1 and SGLT2 have a different affinity and capacity for glucose transport, as well as different expression patterns (Table 2) [41].

**Figure 1:**
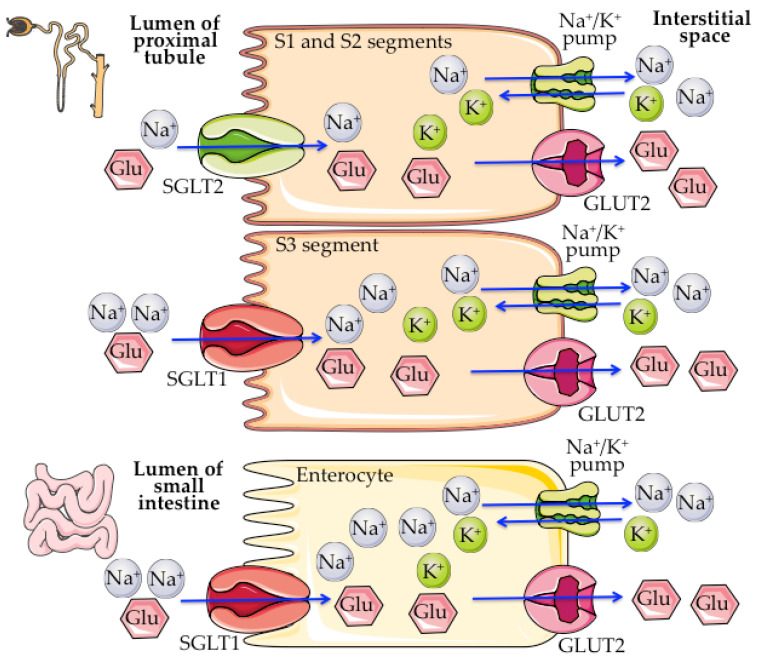
Main sites of expression and function of SGLT1 and SGLT2. Glu: glucose. GLUT2: Facilitated Glucose Transporter Member 2. The figure was drawn using pictures from Servier Medical Art, which are licensed under a Creative Commons Attribution 3.0 Unported License (http://smart.servier.com/, 15 April 2022).

SGLT1/2 mediate sodium-dependent sugar transport driven by the electrochemical sodium gradient created by the sodium/potassium-ATPase, which pumps sodium outside the cell into the bloodstream, creating a sodium gradient between the two sides of the membrane that allows SGLT1/2 to co-transport sodium and sugar from the extracellular side into the cytosol [44] (Figure 1). 

SGLT1 is mainly expressed in the brush border of the small intestine, where it translocates one D-glucose/galactose molecule together with two sodium ions from the diet into mature enterocytes, being responsible for the intestinal absorption of glucose and galactose [45]. Moreover, SGLT1 also behaves as a water and urea channel, which is important for the absorption of water by passive transport in the small intestine [46,47]. SGLT2 is mostly located in the luminal membrane of the S1 and S2 segments of the renal proximal convoluted tubule, co-transporting one D-glucose molecule together with one sodium ion from the glomerular filtrate into the proximal tubule epithelial cells, and being responsible for the reabsorption of ~ 90% of the filtered glucose, while the remaining ~ 10% is reabsorbed by SGLT1 in the S3 segment, where there is less luminal glucose [43]. This exchange alters the glucose concentration inside the cell, being passively transported through the basolateral side of the cell via facilitative glucose transporters (GLUT) into the interstitial space and subsequently to the bloodstream (Figure 1) [43].

In T2DM the capacity to reabsorb renal-filtered glucose seems to be enhanced, which contributes to the increasing levels of glucose circulating in these patients and to the worsening of the disease [48,49]. Whether SGLT2 renal expression can be affected by T2DM remains controversial. Different studies using rodent animal models of T2DM have shown, on the one hand, an increased renal expression of SGLT2 [50,51,52,53,54], and on the other hand, a decreased expression [55,56] compared with non-diabetic animals. As well, in humans, lower [57] and higher [58,59] renal SGLT2 expressions in diabetic patients compared with non-diabetic patients have also been described. Despite the experimental differences among them, taken together, there are more studies in the bibliography supporting the increased expression levels of SGLT2 in diabetes than the contrary. Anyway, SGLT2 inhibition has been established as an effective therapeutic approach to treating T2DM and associated co-morbidities [10].

## 3. SGLT2 Inhibitors 

Phlorizin was the first natural SGLT1/2 inhibitor discovered [8]. Despite its benefits improving insulin resistance [1] and diabetic complications such as nephropathy [23], non-alcoholic fatty liver disease [29], diabetic cardiomyopathy [35], or endothelial dysfunction [34], phlorizin had several drawbacks as a therapeutic agent, which included: (1) low oral bioavailability [60], (2) poor intestinal absorption and rapid clearance in urine [60], (3) low stability, being metabolized to phloretin in the small intestine by β-glucosidases [9], which inhibits GLUT1 [61] (responsible for glucose uptake in many tissues including the brain [62]), and (4) low solubility in water [63]. All of these observations, and the fact that mutations in the SGLT1 gene are associated with intestinal glucose–galactose malabsorption and associated fatal diarrhea [38,64], and that osmotic water flow through SGLT1 is blocked by phlorizin [65], prevented phlorizin from its use as an anti-diabetic drug. This led the scientific community to focus their efforts in the development of other glycoside-based molecules with a higher affinity for the inhibition of SGLT2 (SGLT2i), which could also overcome phlorizin pharmacokinetic limitations.

All synthetic SGLT2i are formed by a sugar head group linked to a long aromatic aglycone tail [9]. The sugar moiety interacts with the glucose binding site and the aglycone part binds to the extracellular vestibule of SGLT2 in the outward-open conformation, interrupting the transport cycle [66]. The first class of synthetic SGLT2i were O-glucosides, such as phlorizin, but they were synthesized as pro-drugs that need to be metabolized in the liver into their active form, avoiding intestinal β-glucosidases degradation [9]. T-1095 was the first synthetic O-glucoside to be developed, T-1095A being its active form [67]. T-1095A was able to inhibit SGLT2-dependent glucose renal reabsorption at the same time that it decreased GLUT2 expression in the kidney, and reduced blood glucose and HbA1c levels in diabetic animal models [67,68]. This finding paved the way for the development of a new class of oral anti-diabetics commonly known as gliflozins, most of them approved for their use in different countries (Table 2). 

After T-1095, other *O*-glucosides were synthesized as pro-drugs, such as sergliflozin [69] or remogliflozin [70]. However, O-glucosides-SGLT2i were still metabolically unstable due to their recognition and cleavage by the β-glucosidases in circulation [9]. Meanwhile, C-glucosides (stable analogs of the corresponding O-glucosides) are metabolically more stable, showing a better oral bioavailability, and have a similar IC_50_ value for SLGT2 inhibition, which is translated into a lower dosage to achieve the same inhibitory effect (100-fold to 3000-fold) [9]. So T-1095 and sergliflozin were discontinued after phase II trials to be replaced for a new generation of C-glucosides, while remogliflozin remains as a low-cost SGLT2i in India despite having to be administrated twice daily [71]. Apart from this exception, all of the SGLT2i approved nowadays for the treatment of T2DM and, in some cases, its associated complications, are C-glucosides: dapagliflozin, canagliflozin, empagliflozin, ipragliflozin, luseogliflozin, tofogliflozin, and ertugliflozin [9]. Particularly, sotagliflozin has been approved in Europe for the treatment of T1DM [72] (Table 3). To note, although SGLT2i are not fully selective for SGLT2 co-transporters and inhibit SGLT1 to a different degree, due to the considerably higher affinity for SGLT2 than for SGLT1 they are commonly referred to as SGLT2i.

The SGLT2i mechanism of action is independent of insulin signaling/sensitivity and seems to overcome the commoner side effects of traditional anti-diabetic drugs, including hypoglycemia, weight gain, cardiovascular risk, gastrointestinal system problems, or even cancer risk [73].

**Table 3:** Main SGLT2i developed, their human SGLT2/1 selectivity calculated by IC_50_ (concentration causing half of the maximal inhibition) SGLT1/SGLT2 and first approved therapeutic use.

Generic Name	Brand Name	Company	SGLT2 Selectivity over SGLT1	First Global Approval	Therapeutic Use
Tofogliflozin	Apleway^®^	Chugai Pharmaceutical Co.	~2912 fold [74]	PMDA-2014 [75]	T2DM [75]
Empagliflozin	Jardiance^®^	Boehringer Ingelheim GmbH. and Eli Lilly and Company	~2600 fold [76]	FDA-2014 [77] FDA-2016 [78] FDA-2021 [79] FDA-2022 [80]	T2DM [77] CV death in T2DM [78] HFrEF [79] Heart failure [80]
Bexagliflozin	-	Theracos Inc.	~2435 fold [81]	Under clinical trials	-
Ertugliflozin	Steglatro™	Merck & Co. and Pfizer Inc.	~2200 fold [82]	FDA-2017 [83]	T2DM [83]
Luseogliflozin	Lusefi^®^	Taisho Pharmaceutical Holdings Co.	~1730 fold [84]	PMDA-2014 [85]	T2DM [85]
Dapagliflozin	Forxiga^®^ (E.U.) Farxiga^®^ (U.S.)	AstraZeneca and Bristol-Myers Squibb Co.	~1200 fold [86]	EMA-2012 [87] FDA-2020 [88] FDA-2021 [89]	T2DM [87] HFrEF [88] CKD [89]
^#^ Remogliflozin	Remozen™	Glenmark Pharmaceuticals Ltd.	~902 fold [70]	CDSCO-2019 [90]	T2DM [90]
^#^ Sergliflozin	-	Glaxo Smith Kline (GSK) Plc.	~300 fold [69]	Discontinued after Phase II [43]	-
Ipragliflozin	Suglat^®^	Astellas Pharma Inc. and Kotobuki Pharmaceutical Co.	~254 fold [91]	PMDA -2014 [92]	T2DM [92]
Canagliflozin	Invokana^®^	Janssen Global Services, L.L.C. and Mitsubishi Tanabe Pharma Co.	~155 fold [93]	FDA-2013 [94] FDA-2018 [95] FDA-2019 [96]	T2DM [94] CV risk in T2DM [95] DKD and risk of hospitalization for HF in T2DM [96]
^#^ T-1095	-	Tanabe Seiyaku Company Ltd.	~59 fold [76]	Discontinued after Phase II [97]	-
Licogliflozin	-	Novartis International A.G.	~35 fold [98]	Under clinical trials	-
Sotagliflozin	Zynquista™	Sanofi-Aventis Group S.A. and Lexicon Pharmaceuticals, Inc.	~20 fold [99]	EMA-2019 [72]	T1DM [72]
^#^ Phlorizin	-	-	~13 fold [76]	-	-

PMDA: Pharmaceuticals and Medical Devices Agency, Japan; FDA: U.S. Food and Drug Administration; CDSCO: Central Drugs Standard Control Organization, India; T2DM: type-2 diabetes mellitus; CV: cardiovascular; EMA: European Medicines Agency; HFrEF: heart failure with reduced ejection fraction; CKD: chronic kidney disease; DKD: diabetic kidney disease. ^#^ O-glucosides, the rest of the compounds are C-glucosides.

Moreover, apart from their direct benefit in reducing glycemia, the greater advantage of SGLT2i is that they have a cardio/renoprotective effect not only in diabetic, but also in non-diabetic patients, although the mechanism remains unclear [65,66,67,68].

Since T2DM is a heterogeneous disease with different associated co-morbidities, and the fact that T2DM patients´ response to the glucose-lowering agents available is quite variable [100], SGLT2i are currently approved for their use in combination with other drugs, complementing their effectiveness to achieve a personalized medicine depending on the clinical characteristics of patients, not only in T2DM, but also in cardio/renal diseases outside the context of T2DM [101,102]. 

**Figure 2:**
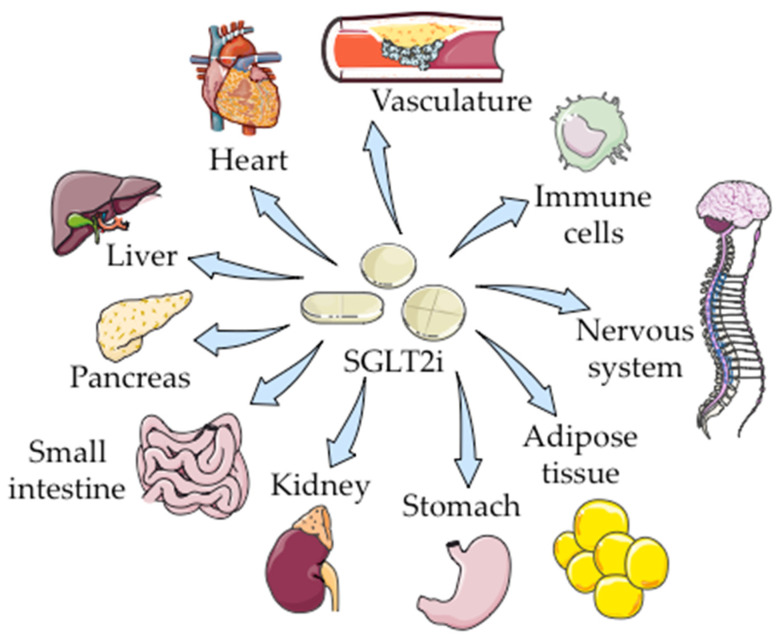
Main therapeutic targets of SGLT2i. The figure was drawn using pictures from Servier Medical Art, which are licensed under a Creative Commons Attribution 3.0 Unported License (http://smart.servier.com/, 21 April 2022).

Although the first interest in SGLT2i was their glucose-lowering capacity, lately there have been described pleiotropic effects of SGLT2i in a wide range of targets throughout the body (Figure 2). Using different experimental approaches and in different scenarios, SGLT2i have been shown to participate in the regulation of osmotic natriuresis and diuresis [103,104], hypertension [105], glucagon [106,107] and energy metabolism [108,109,110,111,112], mitochondrial function and biogenesis [113,114,115,116,117,118], autophagy [112,119,120,121,122,123,124,125], oxidative stress [46,123,126,127,128,129], fibrosis [46,130,131,132,133], apoptosis [122,128,134,135,136,137,138,139], endoplasmic reticulum stress [124,139,140,141,142,143], or inflammation [120,125,126,129,132,137,144,145], which opens a window to explore new uses of these drugs to treat other pathologies.

## 4. SGLT2i and Inflammation

On one hand, as mentioned in the introduction of this review, phlorizin, the first natural SGLT1/2 inhibitor discovered, is a molecule that has anti-inflammatory properties [146,147,148], along with other natural and synthetic chalcones and flavonoids [149,150,151]. On the other hand, the fact that inflammation and metabolism have a strong interplay and that their deregulation drives the development of several diseases is becoming increasingly evident. Normal cellular homeostasis relies on the crosstalk between the immune system and metabolic regulation in such a way that the pathogenesis of metabolic disorders is often triggered by or associated with inflammatory processes [152,153,154,155,156]. In keeping with this, many authors have started to refer to this fact as metaflammation [157,158]. 

**Figure 3:**
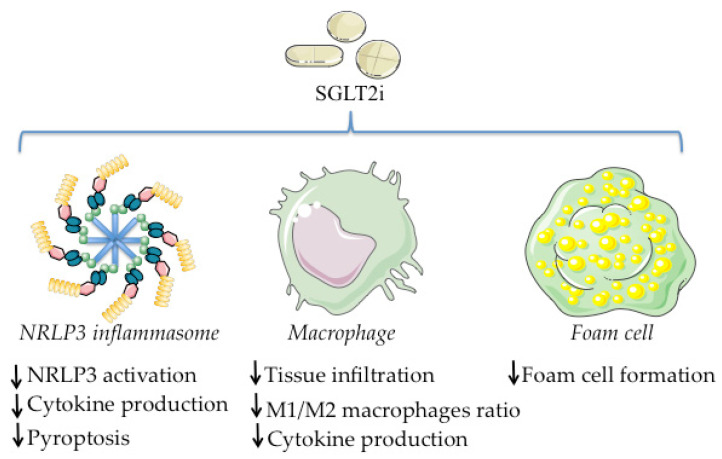
Main anti-inflammatory effects of SGLT2i. The figure was drawn using pictures from Servier Medical Art, which are licensed under a Creative Commons Attribution 3.0 Unported License (http://smart.servier.com/, 21 April 2022).

Taking into account that synthetic SGLT2i are based on the chemical structure of phlorizin, the close interplay between inflammation and metabolic deregulation, and the positive effects of SGLT2i observed in pathologies with metabolic and inflammatory derangement, such as T2DM [159], heart failure [160], diabetic cardiomyopathy [161], diabetic nephropathy [162], or NAFLD [163], it was natural to explore their role as potential therapeutic agents to treat inflammation. Recently, several studies have described the anti-inflammatory effect of SGLT2i using different experimental approaches (Figure 3).

### 4.1. Regulation of Macrophage Tissue Infiltration, Polarization, and Cytokine Production 

Macrophages are resident or infiltrated innate immune cells present in every tissue that are crucial to regulating not only the proper defense against pathogens, but also normal tissue homeostasis and repair [164]. They are characterized by an extraordinary plasticity that allows them to shift from one phenotype to another depending on the surrounding micro-environment (polarization), which also makes them active players that contribute to damage in pathological states of infection and inflammation [165]. According to this plasticity, macrophages are traditionally classified into two subgroups: M1 macrophages (classically activated), which are activated by Th1-type cytokines or bacterial lipopolysaccharides (LPS) and, in turn, produce pro-inflammatory cytokines, and M2 macrophages (alternatively activated), which are activated by Th2-type cytokines and have anti-inflammatory functions [165]. Although a short-term adaptive inflammatory response is necessary for defense and tissue repair, sustained inflammation is often harmful and contributes to disease progression [153]. However, once macrophages are polarized, they can be re-polarized back into M1 or M2 if they are exposed to the proper signals, a quality that has encouraged the scientific community to use them as a therapeutic target [165]. SGLT2i have been proved to be able to modulate M1/M2 macrophage polarization and infiltration in different conditions, with more studies having been carried out for dapagliflozin, canagliflozin, and empagliflozin. 

#### 4.1.1. Dapagliflozin

In macrophages obtained from healthy humans and polarized into M1 with LPS/interferon-gamma (INFγ) in culture, dapagliflozin pre-treatment can diminish the amount of M1 macrophages and increase the number of M2 macrophages, reducing the M1/M2 ratio in both normoglycemic and hyperglycemic environments. Moreover, dapagliflozin is able to block the LPS-induced secretion of the pro-inflammatory cytokines, interleukin (IL) 1β (IL-1β), IL-6, IL-8, and tumor necrosis factor-α (TNFα), as well as able to increase the expression of the toll-like receptor 4 (TLR4) and activate the nuclear factor kappa-light-chain-enhancer of activated B cells (NFκB) in macrophages [145], both of them important regulators of immune and inflammatory responses [166,167]. 

In the cardiovascular system, in infarcted rat hearts, dapagliflozin treatment after infarction increases myocardial M2 macrophage polarization with a concomitant decrease in M1 via acute-phase response factor (STAT3) signaling, a well-known key factor in the polarization of M2, contributing to attenuate cardiac fibrosis [168,169]. In apolipoprotein E-deficient (ApoE^−/−^) mice with T2DM induced with streptozotocin (STZ), dapagliflozin treatment can attenuate atherosclerosis lesions and decrease atherosclerotic macrophage infiltration [170,171], an effect also observed in ApoE^−/−^ mice fed with a high-fat diet [172], in a normoglycemic rabbit model of atherosclerosis [173], in LDL-receptor-deficient (Ldlr^−/−^) mice with a high-fat and high-sucrose diabetogenic diet [174], or in T2DM rats [175]. Moreover, in mice with abdominal aortic aneurysms, dapagliflozin reduces the risk and progression of small aneurysms in part by attenuating aortic wall macrophage infiltration [176]. 

On the other hand, in diabetic ApoE^−/−^ and *db/db* mice with hepatic steatosis, dapagliflozin reduces macrophage infiltration in the liver, contributing to the amelioration of liver fibrosis and inflammation [177,178]. Regarding the kidney, in T2DM *db/db* mice (mutation in the leptin receptor gene), diabetic otsuka long-evans tokushima fatty (OLETF) rats, and a type I diabetic Akita mouse model (AKITA/Slc) with diabetic nephropathy, dapagliflozin decreases macrophage infiltration and the reduction of the gene expression of the pro-inflammatory markers, monocyte chemoattractant protein-1 (MCP-1) and tumor growth factor-β (TGFβ) in the kidney [179,180,181], an effect also observed in mice with non-diabetic proteinuric nephropathy [182]. 

#### 4.1.2. Canagliflozin

In C57BL/6 male mice with lung injury induced by LPS treatment, canagliflozin can inhibit M1 polarization and promote M2 polarization, reducing the M1/M2 macrophage ratio in the lung in vivo, and in bone marrow-derived macrophages in vitro, contributing to reduced inflammation [183]. As well as this, in a mouse model of human nonalcoholic steatohepatitis (NASH), canagliflozin shows benefits reducing liver inflammation and fibrosis by reducing macrophage infiltration and the M1/M2 macrophage ratio, and decreasing NFκB activity, alleviating acute heart injury [184]. In addition, in bone marrow-derived macrophages in culture stimulated with LPS, canagliflozin prevents the gene expression of IL-1β and TNFα, as well as TNFα secretion to the culture medium, associated with a decreased M1/M2 macrophages ratio [184]. 

In mice fed with a high-fat diet, canagliflozin suppresses the obesity-induced accumulation of macrophages along with a decrease in IL-6, and TNFα gene expression in the nodose ganglion and the hypothalamus, and a decrease of TNFα in skeletal muscle [185]. A similar effect is observed in ApoE^−/−^ mice with atherosclerosis, where canagliflozin reduces the number of infiltrated macrophages in the atheroma plaque, which is related to the halting of the progression of the disease [186].

In diabetic New Zealand Obese (NZO, NZO/HlLtJ) mice and mice with T2DM, induced by treatment with nicotinamide and STZ, and by a high-sucrose diet, canagliflozin treatment reduces renal macrophage infiltration and fibrosis [187,188]. Moreover, in the macrophage THP-1 cell line in culture, canagliflozin activates AMP-activated protein kinase (AMPK) and inhibits the expression of the pro-inflammatory cytokines, IL-1β and IL-6, and monocyte chemoattractant protein-1 (MCP-1) [188]. Similarly, in cultured human endothelial cells, canagliflozin can hinder the IL-1β-stimulated secretion of IL-6 and MCP-1 in an AMPK-dependent manner [189].

#### 4.1.3. Empagliflozin

Similar to dapagliflozin and canagliflozin, evidence of empagliflozin effects on macrophage polarization/infiltration in different experimental models exists. Empagliflozin has been shown to unbalance the amount of M1 macrophages in a culture of LPS-stimulated RAW 264.7 macrophages into M2 macrophages by inhibiting IκB kinase (IKK)/NFκB, mitogen-activated protein kinase kinase 7 (MKK7)/c-Jun N-terminal kinases (JNK), and janus kinase 2 (JAK2)/STAT1/3 pathways [190], and by activating AMPK [191].

In the cardiovascular system, empagliflozin reduces the amount of macrophage infiltration in the atheroma plaque and the perivascular adipose tissue in ApoE^-/-^ mice, counteracting atherogenesis and vascular inflammation [192]. In this line, in STZ-diabetic mice, empagliflozin prevents the proliferation of plaque resident macrophages, which contributes to plaque regression [189]. On the other hand, in ApoE^−/−^ mice with abdominal aortic aneurysms induced by angiotensin II, empagliflozin decreases macrophage infiltration in the aortic aneurysms sections [193]. Within the heart, empagliflozin shows early cardioprotective effects in diabetic *db/db* mice, which are in part due to the polarization of M1 macrophages into M2 in the cardiac tissue [194] and the reduction of cardiac macrophage infiltration [195]. In mice and rats with HFrEF, empagliflozin also reduces cardiac macrophage infiltration, contributing to the amelioration of the disease [196,197]. Furthermore, in C57BL/6J mice with corticosterone-induced cardiomyopathy, empagliflozin reduces M1 macrophage infiltration and increases the amount of M2 macrophages in the myocardium [198]. 

In C57BL/6J mice with T2DM and NAFLD induced by STZ and a high-fat diet, empagliflozin reduces the hepatic amount of M1 pro-inflammatory macrophages and induces autophagy in macrophages via activating the AMPK/mammalian target of the rapamycin (mTOR) signaling pathway and inhibiting the IL-17/IL-23 axis (which includes IL-1β, STAT3, or IL-6, among others), contributing to the amelioration of NAFLD [199]. Additionally, in C57BL/6J mice with NAFLD induced by choline-deficient, L-amino acid-defined, high-fat diet, empagliflozin attenuates M1 macrophage activation in the liver [200]. Moreover, in a mouse model of non-alcoholic steatohepatitis and diabetes, empagliflozin decreases the level of macrophage accumulation in the liver [201].

In mice and rats with diabetic nephropathy, empagliflozin can inhibit macrophage infiltration in the renal glomeruli, which is associated with decreased renal injury [202,203]. In line with this, empagliflozin also reduces macrophage infiltration in the kidney in male C57Bl/6N mice with LPS-induced renal injury [204], diabetic *db/db* mice [195], and in rats with renal fibrosis [205].

Regarding the adipose tissue, in ApoE^−/−^ mice fed with a western diet, empagliflozin treatment reduces the amount of adipose tissue, the adipocyte size, and macrophage infiltration, contributing to reducing inflammation in the adipose tissue [206]. In diet-induced-obese mice, empagliflozin treatment alleviates obesity-induced inflammation and insulin resistance by decreasing M1-polarized macrophage accumulation, with the concomitant increase of the anti-inflammatory M2 phenotype, in fat and the liver, which, in part, promotes fat browning [207,208]. 

#### 4.1.4. Other SGLT2i

In concordance with its family members dapagliflozin, canagliflozin, and empagliflozin, luseogliflozin and ipragliflozin also have proved anti-inflammatory effects. In diabetic ApoE^−/−^ mice, luseogliflozin reduces the number of macrophages in the aorta, which correlates with the sizes of the atheroma areas [209], and ipragliflozin has been shown to have a similar effect in the femoral artery [210]. Moreover, ipragliflozin suppresses macrophage foam cell formation in mouse models of type 1 and T2DM and prevents atherosclerotic lesions along with dapagliflozin [171]. On the other hand, in C57BL/6 mice with STZ-induced diabetes, ipragliflozina ameliorates endothelial dysfunction, in part by reducing the amount of infiltrated macrophages in the abdominal aorta [211].

In the perivascular adipose tissue, both luseogliflozin and ipragliflozin decrease macrophage infiltration in mice fed with a high-fat diet and a western diet, respectively, contributing to counteracting adipose tissue remodeling [210,212]. On the other hand, ipragliflozin has been suggested to contribute to healthy adipose tissue expansion in high-fat diet-fed mice by reducing the M1/M2 macrophage ratio within the epididymal adipose tissue [213,214], and in the perirenal adipose tissue [215]. Furthermore, in high-fat diet-fed mice, ipragliflozin reduces the amount of macrophages in the liver and kidney [214,215]. 

### 4.2. Effects on NLRP3 Inflammasome 

The NOD-, LRR-, and pyrin domain-containing protein 3 (NLRP3) inflammasome, the most widely studied inflammasome, is a multiprotein complex of the innate immune system that is mainly activated by pathogen infection and cellular damage. It is made up of a sensor (NLRP3, mainly expressed in macrophages and immune cells [216]), an adaptor (PYCARD, PYD, and CARD Domain Containing), and an effector (caspase 1), which cleaves and activates the pro-inflammatory cytokines pro-IL-1β and pro-IL-18, the end products of NLRP3 inflammasome activation that in turn enhance a pro-inflammatory response causing a cytokine storm [217,218]. On the other hand, caspase-1 activation can also cleavage and activate gasdermin D (GSDMD), the final effector of pyroptosis, a strong inflammatory process that leads to cell death in response to infection [219]. Although NRLP3 inflammasome activation is key to defense against pathogens and removing damaged cells, a deleterious activation of NLRP3 inflammasome and pyroptosis is associated with the pathogenesis of different inflammatory/metabolic diseases, including T2DM [220], cardiovascular disease [221], kidney disease [222], or liver disease [223], and its inhibition has been proved to be an effective therapeutic target [218,219]. 

#### 4.2.1. Dapagliflozin

In diabetic ApoE^−/−^ mice with NASH, dapagliflozin decreases the activation of the NLRP3 inflammasome in the liver, with the concomitant decrease in caspase-1 activity and secretion of IL-1β and IL-18 [177]. In accordance with this, also in ApoE^−/−^ mice with diabetes and atherosclerosis, dapagliflozin treatment reduces circulating levels of NLRP3, IL-1β, and IL-18, as well as in the abdominal aorta, which contributes to reducing the atherosclerotic lesions and to increasing its stability [170]. Similarly, in mice with progressive T2DM (BTBR *ob/ob*), dapagliflozin decreases the myocardial expression of NLRP3, TNFα, and caspase-1, ameliorating the diabetic cardiomyopathy [224]. 

In rats with ulcerative colitis induced by acetic acid, dapagliflozin inhibits the NLRP3/caspase-1 signaling and decreases IL-1β expression in the colon through AMPK-dependent inactivation of NFκB [225]. The same effect is observed in rats subjected to chronic unpredictable stress, in which dapagliflozin reduces serum levels of both IL-1β and IL-18 cytokines, and decreases the expression of p-NFκB, NLRP3, caspase-1, IL-1β, and IL-18 in the hippocampus, suggesting a potential role of dapagliflozin in the treatment of depression [226]. 

In C57BL/6J mice with renal ischemia/reperfusion injury-induced kidney fibrosis, dapagliflozin decreases de expression of NLRP3 and blocks the activation of IL-1β, IL-18, and caspase-1 in the kidney [227]. In accordance with this, in BTBR *ob/ob* mice with diabetic nephropathy, dapagliflozin decreases the expression of NLRP3, TNFα, and caspase-1 in the kidney, along with an increase in AMPK phosphorylation, counteracting the disease progression [228]. 

#### 4.2.2. Canagliflozin

In LPS-treated NIH Swiss male mice, canagliflozin inhibits NLRP3 inflammasome activation, shown by reduced plasma levels of IL-1β and IL-18, and by the reduced production of NLRP3, IL-1β, and IL-18 in the lung, an effect also observed in the mouse monocyte-macrophage cell line J774A.1 with LPS-stimulated NLRP3 activation, where the inactivation of NFκB and the promotion of AMPK phosphorylation induced by canagliflozin treatment seem to be key to blunt NLRP3 activation [229]. 

#### 4.2.3. Empagliflozin

In T2DM patients at high risk of developing cardiovascular diseases, empagliflozin treatment can diminish NLRP3 inflammasome activation and secretion of IL-1β in isolated human macrophages after stimulation with LPS and Adenosine triphosphate (ATP) or palmitate, which could be one of the mechanisms that contribute to the proven empagliflozin´s cardioprotective effects [230]. Likewise, in human aortic smooth muscle cells in culture, empagliflozin can inhibit cell proliferation/migration, NLRP3 expression, caspase-1 activation, and the secretion of the pro-inflammatory markers IL-1β and IL-18 by targeting the signaling of the pro-inflammatory cytokine IL-17A, suggesting a protective role of empagliflozin on the pathogenesis of vascular proliferative diseases [231]. 

In mice with HFrEF, empagliflozin hinders NLRP3 activation by reducing the expression of NLRP3, NFκB, caspase-1, and TNFα, with the subsequent decrease of IL-1β and IL-18 expression in the myocardium, as well as in perfused hearts subjected to ischemia/reperfusion injury and in isolated human cardiomyocytes in culture stimulated with LPS [196]. On the other hand, in non-diabetic mice, empagliflozin protects the heart against doxorubicin cardiotoxicity by reducing the cardiac expression of NLRP3 and myeloid differentiation primary response 88 (MyD88, another central player of the innate immune response [232]), both of them recognized triggers of the cytokine storm involved in heart failure [233].

In mice with obesity and T2DM, the diet-induced activation of the NLRP3 inflammasome in the kidney and liver was blunted by empagliflozin treatment, shown by the reduction in caspase-1 activation and the production of IL-1β, along with lipid accumulation, an effect not observed in the heart despite the efficient reduction of cardiac lipid accumulation [234]. 

In pancreatic β TC-6 cells, an insulin-secreting cell line derived from transgenic mice expressing the large T-antigen of simian virus 40 (SV40) in pancreatic β-cells, empagliflozin treatment hinders the high-glucose-induced expression of NLRP3, caspase-1, and GSDMD, an effect also observed in vivo in the pancreas of diabetic mice that was associated with the prevention of pathological changes in pancreatic tissues [235]. In keeping with this, in *db/db* mice with a reduced ejection fraction and cardiac hypertrophy, empagliflozin also reduces NLRP3 and caspase-1 as well as the production of IL-1β and GSDMD, blocking cardiomyocyte pyroptosis in the diabetic heart [236].

## 5. Conclusions

The growing evidence linking the deregulation of inflammation with the triggering and maintenance of many diseases have opened new avenues for treatment by blocking this process. Understanding the mechanism through which this deregulation occurs is crucial for looking for effective therapeutic targets that could, at least, ameliorate the disease. SGLT2i have arrived in the clinical practice with strong evidence supporting their benefits, not only regarding glucose-lowering or cardiorenal outcomes but also in a wide range of tissues and pathologies. Taking into account the research carried out in recent years, it seems clear that SGLT2i have an effect protecting cells from inflammation, a process that could be involved in the pleiotropic beneficial effects of SGLT2i along with the improvement of energy metabolism, and that opens new lines of research regarding the potential role of SGLT2i as anti-inflammatory drugs. 

## Figures and Tables

**Table 2:** Comparison between SGLT1 and SGLT2 co-transporters [42,43].

	SGLT1	SGLT2
Glucose affinity	High (K_m_~0.5–2 mM)	Low (K_m_~2–5 mM)
Glucose transport capacity	Low (2 nmol/mg·min)	High (10 nmol/mg·min)
Renal expression	S3 segment	S1 and S2 segments
Renal glucose reabsorption	3–10%	90–97%
Na^+^/glucose stoichiometry	2:1	1:1
Expression in the small intestine	Yes	No
Main function	Intestinal absorption of glucose and galactose (urea and water)	Renal reabsorption of glucose
